# Improvement of Myocardial Cell Injury by miR-199a-3p/mTOR Axis through Regulating Cell Apoptosis and Autophagy

**DOI:** 10.1155/2022/1642301

**Published:** 2022-06-22

**Authors:** Weixiong Wu, Xingfeng Chen, Qingyang Hu, Xuefei Wang, Jingyu Zhu, Qianzhen Li

**Affiliations:** ^1^Department of Cardiovascular Surgery, Fujian Medical University Union Hospital, No 29 Xinquan Road, 350001 Fuzhou, China; ^2^Fujian Key Laboratory of Vascular Aging, Fujian Medical University, No 29 Xinquan Road, 350001 Fuzhou, China; ^3^Key Laboratory of Cardio-Thoracic Surgery, Fujian Medical University, No 29 Xinquan Road, 350001 Fuzhou, China

## Abstract

**Background:**

Myocardial ischemia-reperfusion injury (MIRI) is characterized by its high incidence rate and mortality. miR-199a-3p is thought to be strongly linked with the development of some myocardial diseases, but the influence of miR-199a-3p in MIRI remains unclear.

**Methods:**

AC16 cells were used. The concentrations of mammalian target of rapamycin (mTOR), light chain 3 II/light chain 3 I, and Beclin-1 were detected with western blotting and qRT-PCR. The binding site between mTOR and miR-199a-3p was evaluated via luciferase report assay. Cell apoptosis was evaluated through flow cytometry.

**Results:**

Knockdown of miR-199a-3p accelerated the myocardial cell injury after L-oxygen treatment. Increased expression of mTOR and suppressed autophagy were observed after knockdown of miR-199a-3p. Knockdown of miR-199a-3p or overexpression of mTOR greatly aggravated cell injury through inhibiting autophagy*. Conclusions.* This study might be helpful for the therapeutic method of MIRI through by regulating miR-199a-3p/mTOR.

## 1. Introduction

Myocardial ischemia-reperfusion injury (MIRI) is characterized by more serious structural injury and metabolic dysfunction than before reperfusion [1], and even irreversible cardiac muscle injury appear in MIRI [2]. MIRI could be found in the pathological process of various heart diseases and operations, including after revascularization of myocardial infarction (MI), after cardiac surgery, or restoring blood supply after heart transplantation [3]. Exploring the potential pathological mechanism is helpful for the prevention and treatment of MIRI.

MicroRNA (miRNA) could affect certain proteins by inhibiting mRNA translation [4], degrading mRNA, or complementary binding with target mRNA [5]. Several miRNAs including miR-663b, miR-17-5p, and miR-451a [6] are closely related to ischemic heart disease [7]. miR-199a-3p was believed to be linked to myocardial repair [8]. Single myocardial injection of miR-199a-3p can improve cardiac function after MI, which confirms that miR-199a-3p acts a key role in myocardial repair [8]. In addition, after ischemia reperfusion, miR-199a-3p could modulate the survival of cardiomyocytes [9], and miR-199a-3p can induce myocardial regeneration [10].

Mammalian target of rapamycin (mTOR) is an atypical and conserved serine/threonine protein kinase, which mainly acts a role through mTOR complex 1 (mTORC1) [11]. The inhibitor of mTORC1 can protect cardiomyocytes by reducing cell energy consumption and activating autophagy, which is the self-protective mechanism of myocardial ischemia. Autophagy is the initial process of cell decomposition and degradation [12]. It acts a key role in maintaining the most basic energy and nutritional needs of cells. In the state of myocardial ischemia and energy deficiency, mTORC1 could be inhibited [13]. Then, the level of cardiomyocyte autophagy is upregulated, and cardiomyocyte death is reduced [14]. Some studies have proved that the AMPK pathway can directly or indirectly regulate mTORC1 in ischemic state and finally cause the upregulation of autophagy level and protect myocardium. However, if miR-199a-3p could regulate MIRI through targeting mTOR remains unclear.

In this study, the MIRI cell model was established with low oxygen (L-oxygen) condition. mTOR overexpression vector (mTOR^OE^) and knockdown and overexpression vectors of miR-199a-3p were constructed. The influence of them on apoptosis and autophagy of cells induced by L-oxygen treatment were measured. This research might unfold the modulating effect of miR-199a-3p in preventing and treating MIRI.

## 2. Methods and Materials

### 2.1. Cell Culture

Cardiomyocytes (AC16, Tongpai biotechnology, Shanghai, China) was chosen in this research for *in vitro* study. DMEM containing 1% penicillin and 5% FBS (Gibco, US) was applied for cell culture. The incubator with 37°C and with CO_2_ condition were used for cell culture. After reaching 70% confluence, cells were used for different experiments.

### 2.2. Cell Transfection

mTOR overexpression vector (mTOR^OE^), knockdown and overexpression vectors of miR-199a-3p, and related control vectors were designed and constructed by GenePharma Co., Ltd. (Shanghai, China). Lipofectamine 2000 was applied in this research for cell transfection. The concentrations of miR-199a-3p inhibitor, mTOR^oe^, and related vectors were 50 nM.

### 2.3. Low Oxygen Treatment (L-Oxygen)

The L-oxygen cell model was established with oxygen and glucose deprivation/reoxygenation method. Cells were cultured with medium free of serum for 24 h. Then, the hypoxia cell model was established by incubating cells at 37°C with 5% CO_2_ and 1% O_2_. Cells were subsequently cultivated on the condition of 95% air and 5% CO_2_ (24 h).

### 2.4. Western Blotting

Cells were lysed with radio immunoprecipitation assay (RIPA) lysate with 1% phenylmethanesulfonyl fluoride (PMSF). Bicinchoninic acid (BCA) commercial kit was used to measure protein content. 10% sodium dodecyl sulfate-polyacrylamide gel electrophoresis (SDS-PAGE) was performed. After transferring to the PVDF membrane, which was blocked using 10% skimmed milk (2 h). The PVDF membranes were cultured with primary antibodies overnight at 4°C. The PVDF membranes were cultured with secondary antibody for 4 h. The proteins were measured with chemiluminescence with Thermo ECL Substrate (Bio-Rad). The protein bands were analyzed using ImageJ software.

### 2.5. Reverse Transcription Polymerase Chain Reaction (RT-PCR)

RNA was firstly extracted using TRIzol reagent, and SuperScriptTM II Reverse Transcriptase was applied to reverse-transcribe extracted RNA into cDNA. RT-PCR was conducted with SYBR Premix Ex TaqTM II kit. The primers were listed in Table 1. The target genes mRNA levels were detected with *ΔΔ*Ct method.

### 2.6. Flow Cytometry

After different treatments, trypsin was used to digest cells. Annexin V-FITC and propidium iodide (Beyotime, Beijing, China) were used to incubate cell in the dark for 30 min. Finally, the flow cytometry method was applied for cell apoptosis detection.

### 2.7. Dual Luciferase Reporter Assay

Cell transfection was conducted with Lipofectamine 2000 (Invitrogen life technologies, USA). 2 days later, dual-luciferase reporter gene assay kit was applied to evaluate luciferase activity.

### 2.8. Statistical Analysis

The results were shown with mean ± SD. SPSS software was used for data analysis. One-way ANOVA was applied for analyzing differences between different groups. *P* < 0.05 suggests statistical difference.

## 3. Results

### 3.1. Knockdown of miR-199a-3p Accelerated the Myocardial Cell Injury after L-Oxygen Treatment

**Figure 1:**
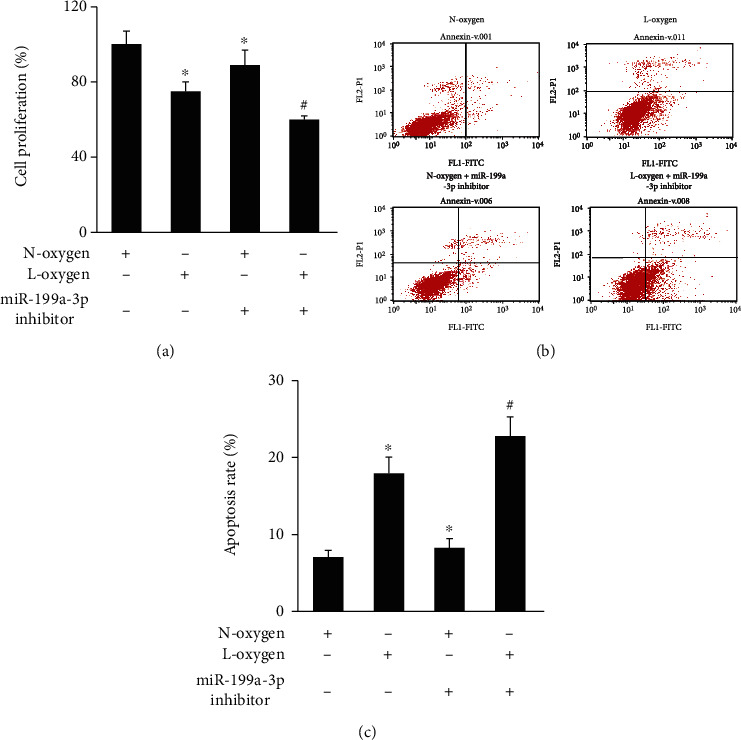
miR-199a-3p inhibitor accelerated the myocardial cell injury after L-oxygen treatment. (a) The cell proliferation was measured after L-oxygen, N-oxygen, or miR-199a-3p inhibitor administrations. (b) The cell apoptosis was measured after L-oxygen, N-oxygen, or miR-199a-3p inhibitor treatment. (c) Cell apoptosis was quantified. ^∗^*P* < 0.05 compared to group N-oxygen. ^#^*P* < 0.05 compared to group L-oxygen.

The myocardial cell injury model was established through L-oxygen treatment, and the influence of knocking down miR-199a-3p on cell viability and apoptosis of cardiomyocyte was evaluated. L-oxygen treatment significantly inhibited cell proliferation and promoted apoptosis (Figures 1(a)–1(c)). In addition, supplementary treatment with knocking down miR-199a-3p remarkably aggravated the effects of L-oxygen. Significant decrease of cell viability and increased of cell apoptosis were found in the group L-oxygen+ miR-199a-3p inhibitor (Figures 1(a)–1(c)).

### 3.2. miR-199a-3p Inhibitor Increased the Level of mTOR and Suppressed Autophagy

**Figure 2:**
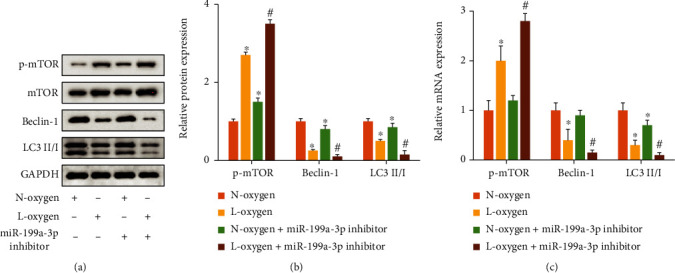
miR-199a-3p suppressor elevated the concentration of mTOR and restrained autophagy. (a) The protein expression levels were evaluated via western blotting. (b) The protein expression levels were quantified. (c) The mRNA expression levels were evaluated via qRT-PCR. ^∗^*P* < 0.05 compared to group N-oxygen. ^#^*P* < 0.05 compared to group L-oxygen.

The expression of phosphorylated mTOR (p-mTOR) was increased, but light chain 3 II/light chain 3 I (LC3 II/I) and Beclin-1 were suppressed in the group L-oxygen compared to group N-oxygen (Figures 2(a)–2(c)). In addition, knockdown of miR-199a-3p remarkably promoted p-mTOR expression but inhibited Beclin-1 and LC3 levels on the condition of both L-oxygen and N-oxygen (Figures 2(a)–2(c)).

### 3.3. Overexpression of mTOR or miR-199a-3p Inhibitor Remarkably Aggravated Cell Injury

**Figure 3:**
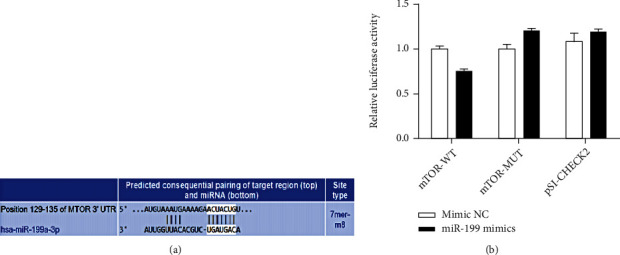
Binding sites between miR-199a-3p and mTOR was identified. (a) Binding site between miR-199a-3p and mTOR was predicted with bioinformatics method. (b) Binding site between miR-199a-3p and mTOR was identified. ^∗^*P* < 0.05 compared to the group mimic NC.

**Figure 4:**
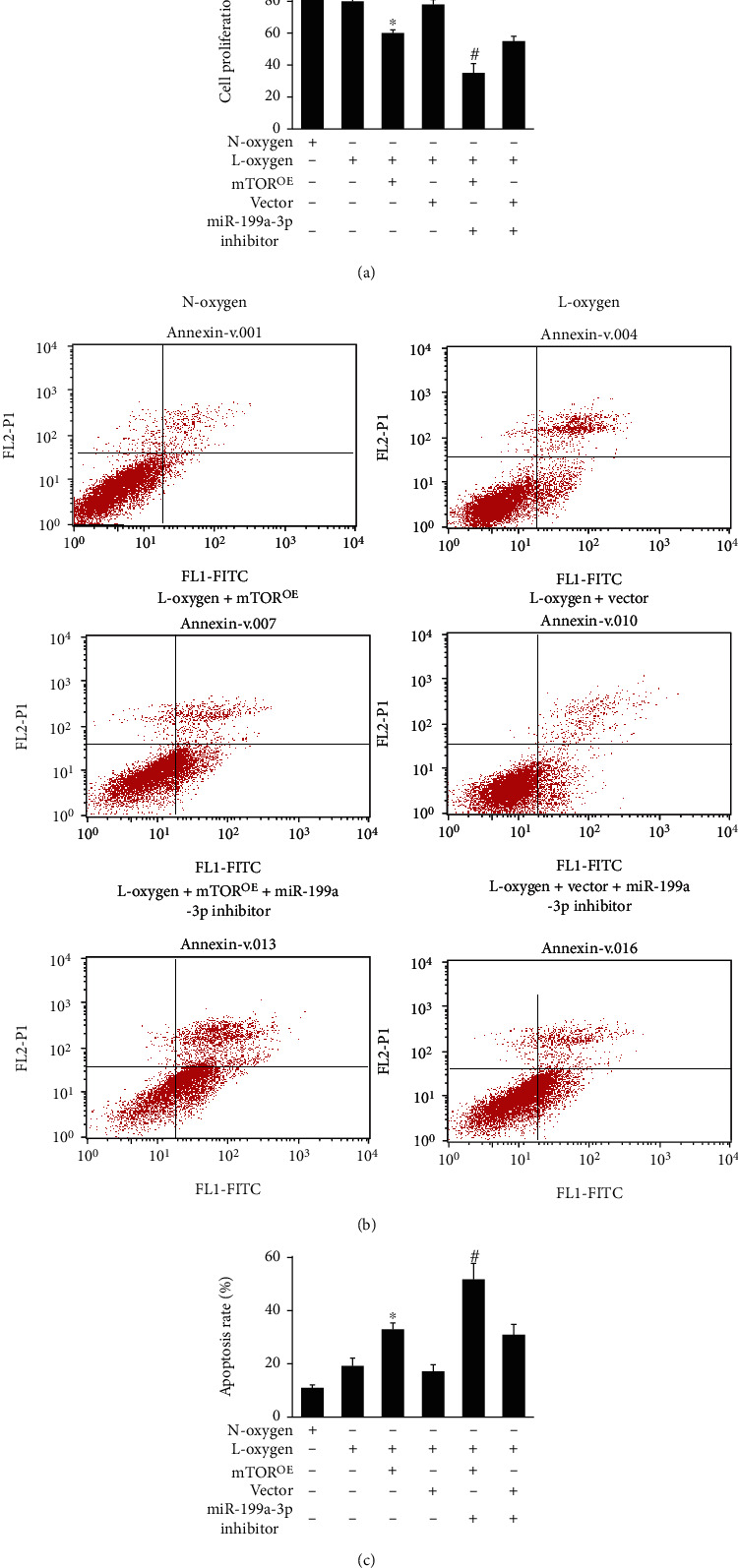
Overexpression of mTOR or miR-199a-3p inhibitor greatly aggravated cell injury. (a) The cell proliferation was measured after different treatments. (b) Flow cytometry was applied to evaluate cell apoptosis. (c) Cell apoptosis was quantified. ^∗^*P* < 0.05 compared to group L-oxygen. ^#^*P* < 0.05 compared to group L-oxygen+mTOR^OE^.

The binding site was identified successfully with luciferase report assay (Figures 3(a) and 3(b)). Overexpression vector of mTOR was successfully constructed. Overexpression of mTOR could greatly suppress cell viability and promoted apoptosis (Figures 4(a)–4(c)) compared with group L-oxygen+vector. Meanwhile, supplementary treatment with knocking down miR-199a-3p remarkably strengthened the function of mTOR^OE^. Remarkable decrease of cell viability and increased of apoptosis were observed in the group L-oxygen+ mTOR^OE^+miR-199a-3p inhibitor in comparison with group L-oxygen+mTOR^OE^ (Figures 4(a)–4(c)).

### 3.4. Overexpression of mTOR Significantly Inhibited Autophagy

**Figure 5:**
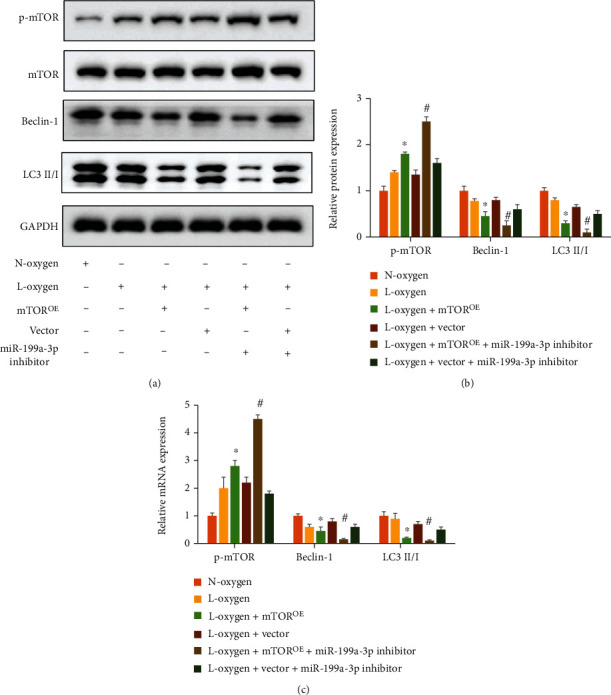
Overexpression of mTOR or miR-199a-3p inhibitor markedly inhibited autophagy. (a) The protein expression levels were evaluated via western blotting. (b) Protein levels were quantified. (c) The mRNA expression was detected with qRT-PCR. ^∗^*P* < 0.05 compared to group L-oxygen. ^#^*P* < 0.05 compared to group L-oxygen+mTOR^OE^.

The influence of mTOR^OE^ and knockdown of miR-199a-3p on autophagy-linked proteins were also investigated. The protein and mRNA levels of p-mTOR were increased, but Beclin-1 and LC3 II/I were suppressed after overexpression of mTOR compared with group L-oxygen (Figures 5(a) and 5(c)). In addition, of miR-199a-3p inhibitor significantly promoted p-mTOR level but suppressed LC3 II/I and Beclin-1 compared to group L-oxygen+ mTOR^OE^ (Figures 5(a)–5(c)).

## 4. Discussion

MIRI is characterized by high incidence rate and mortality in the cardiovascular diseases [3]. Although, new treatment methods (thrombolysis, percutaneous angioplasty, percutaneous coronary intervention, and cardiac bypass) have been greatly improved. The reperfusion injury still cannot be completely solved [15]. MIRI process might be involved in inflammatory factor infiltration, calcium overload, intracellular pH change, and aerobic free radical injury [16, 17]. Unfolding the potential pathological mechanism of MIRI might be helpful to develop new treatment for MIRI.

In the process of autophagy, the transformation of LC3-II is an important step of autophagosomes [18]. In this present study, overexpression of mTOR or knockdown of miR-199a-3p could significantly inhibit autophagy process by suppressing Beclin-1 and LC3II/I ratio (Figures 5(a)–5(c)). These data indicated that regulation of mTOR or miR-199a-3p might be the potential function targets treating MIRI.

miRNAs can regulate most biological processes, including autophagy and apoptosis. miRNAs could regulate the courses of some cardiovascular diseases [9, 19]. Previous research indicated that miR-199a-3p modulated myocardial hypertrophy, but the regulatory function of miR-199a-3p in MIRI has not been fully unfold. In this study, the inhibition of autophagy by knocking down miR-199a-3p was demonstrated (Figures 5(a)–5(c)).

In the normal growth state of cells with sufficient growth factors and ATP [20], mTOR could downregulate autophagy signal transduction pathway and maintain a low basic level of autophagy [21]. However, in the state of starvation, autophagy is activated by PI3K-AMPK/mTOR axis. The influence of the PI3K-AMPK/mTOR signaling pathway on MIRI is important and complicated and needs to be further explored.

## 5. Conclusions

In this study, we demonstrated that overexpression of mTOR or miR-199a-3p inhibitor greatly inhibited autophagy but accelerated apoptosis. The modulating effects of miR-199a-3p/mTOR axis in MIRI was identified.

## Figures and Tables

**Table 1:** Primer sequences used in this study.

Gene	Forward	Reverse
LC3	GACCGCTGTAAGGAGGTGC	AGAAGCCGAAGGTTTCTTGGG
mTOR	TCGGTGCAAACCTACAGAAGC	TGCAGGTCGTATATGGACAGAG
GAPDH	ACAACAGCCTCAAGATCATCAG	GGTCCACCACTGACACGTTG
Beclin-1	ATGGAGGGGTCTAAGGCGTC	TGGGCTGTGGTAAGTAATGGA

## Data Availability

The datasets used and analyzed during the current study are available from the corresponding author on reasonable request.

## Abbreviations

MIRI: Myocardial ischemia-reperfusion injury
mTORC1: mTOR complex 1
mTOR^OE^: mTOR overexpression vector
L-oxygen: Low oxygen
N-oxygen: Normal oxygen.
